# Correlation between mesenteric fat thickness and serum apolipoproteins in patients with peripheral arterial occlusive disease

**DOI:** 10.1186/1476-511X-11-125

**Published:** 2012-10-01

**Authors:** Apostolos Perelas, Vanessa Safarika, Ioannis S Vlachos, Irene Tzanetakou, Laskarina-Maria Korou, Panagiotis Konstantopoulos, Ilias Doulamis, Ioannis Ioannidis, Ioannis Kornezos, Dimitrios Gargas, Christos Klonaris, Despina N Perrea, Achilleas Chatziioannou

**Affiliations:** 1 Laboratory for Experimental Surgery and Surgical Research "N.S. Christeas", University of Athens Medical School, 15b Agiou Thoma Street, Goudi, Athens, 11527, Greece; 2 Department of Interventional Radiology, Aretaieion University Hospital, University of Athens Medical School, 15b Agiou Thoma Street, Goudi, Athens, 11527, Greece; 3 Vascular Division, 1st Department of Surgery, Laikon Hospital, University of Athens Medical School, 15b Agiou Thoma Street, Goudi, Athens, 11527, Greece

**Keywords:** Ultrasound, Mesenteric fat thickness, Adipose tissue, Apolipoproteins, Atherosclerosis

## Abstract

**Background:**

Visceral fat possesses the most detrimental potential for cardiovascular morbidity through the release of adipokines, as well as metabolic and proinflammatory mediators, which adversely affect metabolic and vascular homeostasis. Among the different types of visceral adipose tissue, mesenteric fat is considered particularly detrimental, due to its close proximity to the portal circulation, affecting directly the liver, which is the main regulator of body metabolic homeostasis. Mesenteric fat can be reliably estimated using abdominal ultrasonography, the only available imaging method able to depict individual mesenteric leaves. Aim of the present study was to investigate the correlation of mesenteric fat thickness (MFT) with serum apolipoprotein levels in patients undergoing digital subtraction angiography in a single center.

**Methods:**

35 male patients with peripheral arterial disease were examined. After careful examination of the periumbilical area, the mesenteric leaves were identified. The maximal distance between each pair of sequential leaves was measured, and the mean value of the three thickest leaves was determined as the mesenteric fat thickness. Six apolipoprotein fasting serum concentrations were measured using a Luminex proteomics platform (xMAP Multiplex immunoassay): apolipoprotein A-I (apoAI), apolipoprotein A-II (apoAII), apolipoprotein B (apoB), apolipoprotein C-II (apoCII), apolipoprotein C-III (apoCIII) and apolipoprotein E (apoE).

**Results:**

MFT correlated with apoAII and apoB serum concentrations. The correlations with apoAII and apoB remained significant following correction for BMI. No correlations were noted between MFT and serum apoAI, apoCII, apoCIII or apoE levels before or after adjustment for BMI.

**Conclusions:**

Our study indicates that MFT is significantly correlated with the concentration of atherogenic low density lipoproteins particles, as well as with apoAII, a determinant of free fatty acids levels. No correlation was observed between mesenteric fat thickness and very low density lipoprotein or chylomicron particles concentration.

## Background

Obesity has emerged as a global pandemic, recently characterized by the World Health Organization as *globesity*[1]. Its rates are increasing worldwide, with almost 25% and 10% of the global population being overweight and obese, respectively [2]. Obesity has been linked to a large number of diseases, including diabetes mellitus, cardiovascular diseases and numerous forms of cancer [3].

There is an increasing amount of evidence demonstrating that regional rather than total adiposity is a more significant factor for obesity related disorders. Visceral fat possesses the most detrimental potential for cardiovascular morbidity through the release of adipokines, as well as metabolic and proinflammatory mediators, which adversely affect metabolic and vascular homeostasis [4]. Among the different types of visceral adipose tissue, mesenteric fat is considered particularly detrimental, due to its close proximity to the portal circulation, affecting directly the liver, which is the coordinator of body metabolic regulation [5].

Mesenteric fat can be reliably estimated using abdominal ultrasonography, the only available imaging method able to depict individual mesenteric leaves [5]. Until today, mesenteric fat has been correlated with metabolic and cardiovascular markers, such as blood lipoprotein concentrations [5]. However, there are no available studies investigating the possible influence of mesenteric fat to specific apolipoproteins, constituents of serum lipoproteins and key molecules to their function.

Apolipoprotein A-I (apoAI) is the major structural component of high density lipoprotein (HDL), exhibiting significant atheroprotective functions [6]. Apolipoprotein A-II (apoAII) is the second most important protein component of HDL and seems to have a significant role in triglyceride metabolism [7]. Its levels have been correlated with fat distribution in humans [7]. Apolipoprotein B (apoB) is the major protein constituent of the atherogenic low density lipoprotein (LDL), intermediate density lipoprotein (IDL), very low density lipoprotein (VLDL) and of chylomicrons [6]. It has been proposed that serum apoB concentration may be a more reliable marker than serum LDL determination, since it can be directly measured, rather than estimated through a relevant formula [6]. Apolipoprotein C-II (apoCII) is a protein component of VLDL and contains a cofactor for lipoprotein lipase, which hydrolyzes triglycerides into fatty acids [8]. In humans, hypertriglyceridemia is regularly accompanied by a significant increase in apoCII plasma levels. Apolipoprotein C-III (apo CIII) is an inhibitor of lipoprotein lipase and of triglycerides remnant uptake by hepatic lipoprotein receptors [9], as well as a component of chylomicrons, VLDL, IDL and LDL. Its levels have been show to correlate with the degree of insulin resistance, while apoCIII overexpression has been found to accelerate the progress of atherosclerosis in rodents [9]. Finally, apolipoprotein E (apoE) is the main ligand for clearance of VLDL and chylomicron remnants, affecting the circulating concentrations of lipoproteins and plasma levels of cholesterol and triglycerides [10].

Aim of the present study was to investigate the correlation of mesenteric fat thickness (MFT) with serum apolipoprotein levels in patients undergoing digital subtraction angiography in a single center. Specifically, 6 apolipoprotein serum concentrations were measured using a Luminex proteomics platform: apoAI, apoAII, apoB, apoCII, apoCIII and apoE.

## Results

The patient group (n = 35) was comprised of 25.7% (n = 9) women and 74.3% (n = 26) men. Mean participant age was 65.5 ± 10.0 years. All patient characteristics (n = 35) are depicted in Table 1.

**Table 1:** Patient characteristics

	
**Parameter**	**Frequency (%)**
Male sex	26 (74.3%)
Smoking	28 (80%)
Dyslipidemia	20 (57%)
Hypertension	26 (74.3%)
**Parameter**	**Mean ± SD**
Age (years)	64.4 ± 10.0
Weight (kg)	78.0 ± 9.7
BMI (kg/m^2^)	26.8 ± 2.7
Total Cholesterol (mg/dl)	205.7 ± 67.5
LDL Cholesterol (mg/dl)	134.5 ± 68.6
Triglycerides (mg/dl)	166.4 ± 82.6
Apolipoprotein A-I (mg/dl)	179.3 ± 50.1
Apolipoprotein A-II (mg/dl)	35.5 ± 18.2
Apolipoprotein B (mg/dl)	76.3 ± 50.2
Apolipoprotein C-II (mg/dl)	6.9 ± 4.2
Apolipoprotein C-III (mg/dl)	19.8 ±10.6
Apolipoprotein E (mg/dl)	5.8 ± 2.4
Mesenteric Fat Thickness (cm)	0.73 ± 0.41

Mean MFT was 0.73 ± 0.4 cm. There were no statistically significant differences in MFT between the two sexes, even after correction for BMI and BMI and age (Table 2).

**Table 2:** Comparison of mesenteric fat thickness (in mm) between patient subgroups

			
**Grouping Parameter**	**Males**	**Females**	**Significance Level**
Sex	0.72 ± 0.43 (n = 26)	0.76 ± 0.4 (n = 9)	0.62 (NS)
**Grouping Parameter**	**Group (+)**	**Group (−)**	**Significance Level**
Hypertension	0.71 ± 0.4 (n = 24)	0.84 ± 0.5 (n = 11)	0.59 (NS)
Dyslipidemia	0.7 ± 0.37 (n = 20)	0.82 ± 0.52 (n = 15)	0.77 (NS)
Previous Angioplasty	0.74 ± 0.5	0.74 ± 0.42	0.85 (NS)

*NS* Non Significant.

In a multivariate analysis, no significant differences were detected in MFT measurements between patient subgroups, formed based on previous angioplasty, hypertension and presence of dyslipedemia, even after correction for BMI and BMI and age (Table 2). MFT correlated with apoAII and apoB serum concentrations (Table 3). The correlations with apoAII and apoB remained significant following correction for BMI (Table 3). No correlations were noted between MFT and serum apolipoproteins apoAI, apoCII, apoCIII or apoE before or after adjustment for BMI (Table 3).

**Table 3:** Correlation between MFT and Serum apolipoprotein levels

						
**Correlation with MFT**	**apoAI**	**apoAII**	**apoB**	**apoCII**	**apoCIII**	**apoE**
Correlation Coefficient (Significance Level)	0.28 (NS)	r = 0.5 (p < 0.01)	r = 0.47 (p < 0.01)	−0.06 (NS)	−0.04 (NS)	0.06 (NS)
**Correlation with MFT (correctedfor BMI)**	**apoAI**	**apoAII**	**apoB**	**apoCII**	**apoCIII**	**apoE**
Correlation Coefficient (Significance Level)	0.28 (NS)	r = 0.45 (p < 0.01)	r = 0.44 (p < 0.05)	−0.08 (NS)	−0.07 (NS)	0.06 (NS)

*NS* Non significant.

## Discussion

Obesity, defined by the WHO as BMI > 30, is considered as one of the major preventable causes of death worldwide and is characterized by a positive disequilibrium between energy intake and energy expenditure [11]. It is directly involved in the pathogenesis of insulin resistance, atherosclerosis, dyslipidemia, hypertension and degenerative joint disease [2]. Research conducted in the past few decades has revealed the pleiotropic effects of adipose tissue on every aspect of bodily functions and homeostasis, including immunity, metabolism and aging [12].

Mesenteric fat can be accurately and reliably assessed by ultrasonography, using the methodology described by Liu *et al.*[13]. This method requires significant training by experienced physicians [14]. However, if properly trained, the intra- and inter- operator variability is low [13,15-17]. The presence of obesity does not negatively affect the reliability of the imaging modality, since increased adipose tissue accumulation renders mesenteric leaves readily visible [18].

The estimation of MFT ultrasonographically is one of the newest additions to the array of available imaging techniques for adipose tissue estimation. MFT has demonstrated positive associations with indices of insulin resistance (ie fasting blood glucose, HOMA-IR index, fasting serum insulin) [13,17,19] and subclinical atherosclerosis (carotid intima-media thickness) [13,15]. Increased MFT has been proposed as a significant prognostic factor for polycystic ovary syndrome and fatty liver, conditions directly linked to insulin resistance, the metabolic syndrome, as well as atherosclerosis [16,19].

Even though MFT has been correlated to LDL and HDL serum concentrations [13,17], no studies have been carried out regarding the relationship between MFT and lipoprotein subfractions or serum concentrations. To our knowledge, this is the first available study to investigate the correlation of MFT to a large array of apolipoproteins.

Much attention has been devoted to the effect of apolipoproteins on the progression of atherosclerosis [6]. They act as cofactors for enzymes involved in lipid metabolism, ligands for receptors or mediators of reverse cholesterol transport. Assessment of apolipoprotein levels is useful in predicting risk of cardiovascular disease [6].

Mesenteric fat may have a significant effect on serum lipoproteins by modulating liver function. It is the only adipose tissue depot that drains to the portal circulation, thus directly affecting liver metabolism and biosynthetic activity. It promotes liver fat accumulation and insulin resistance, which in turn leads to elevated hepatic glucose production and increased release of free fatty acids (FFAs) from adipose tissue [20,21].

Abdominal fat cell weight has been previously reported to negatively correlate with plasma apoAI concentration [22]. However, in our study we did not observe a statistically significant relationship of MFT with apoAI levels. On the other hand, MFT measurements correlated linearly with apoAII serum concentrations. This correlation may reflect the role of mesenteric fat as a significant source of triglycerides in the fasting state. A study investigating the impact of weight loss on HDL apoAII kinetics in the metabolic syndrome reported a significant correlation between changes in apoAII fractional catabolic rate and visceral adipose tissue mass, indicating a potential role for adiposity in the regulation of apoAII catabolism [23].

In our study, we also observed a significant correlation between MFT and apoB levels, the major component of LDL lipoproteins. FFAs released by mesenteric fat have been shown to increase apoB production and VLDL release. VLDL is transformed into IDL and LDL, a process, which results in the production of atherogenic LDL lipoproteins containing apoB [24-26]. Mesenteric adipose tissue may be a significant source of FFAs in both the fasting and fed period, given its relative resistance to the lipotrophic effects of insulin and its sensitivity to the lipolytic effects of catecholamines [4,27]. These effects may be exaggerated in individuals with increased mesenteric adipose tissue depots.

As far as the other secretory products of mesenteric fat are concerned, interleukin- 6 and tumor necrosis factor alpha have been shown to increase apoB and VLDL production by hepatocytes in both clinical and experimental studies [25,28-31].

On the other hand, no statistically significant correlations were observed between mesenteric fat thickness and components of VLDL lipoproteins, such as apoCII or apoCIII. This lack of association may be attributed to the design of the study. During the fasting state, VLDL lipoprotein levels tend to be diminished, and this may interfere with the possible relationship between mesenteric adipose tissue and liver lipoprotein production. In a previous study and in accordance to our results, serum apoE levels did not differ between obese and non-obese individuals [32]. However, visceral fat accumulation significantly correlated with serum apoE concentration in middle-aged non-obese Japanese men [33].

The role of mesenteric fat on inflammation and oxidative damage in vascular tissue and the correlation of its thickness with cardiovascular disease risk are documented in literature [34]. Trying to extend the existing knowledge, our study showed that MFT significantly correlates with apoB and apoAII levels. The correlation with apoB confirms the close association between MFT and atherosclerotic risk, observed in a significant number of previous studies. The correlation of MFT with apoAII may reflect the role of mesenteric fat as a significant source of triglycerides in the fasting state.

## Conclusion

Our study indicates that MFT significantly correlates with the concentration of atherogenic LDL apoB particles, as well as with apoAII, a determinant of free fatty acids levels. Our data are in concordance to that of previous studies and demonstrate the significance of visceral adipose tissue as an atherosclerosis risk factor. Further investigation of the pathogenic and regulatory pathways of visceral obesity that promote atherosclerotic procedures is needed and may reveal new targets for atherosclerosis prevention and treatment.

## Methods

Patient population consisted of individuals with peripheral vascular disease (PVD) admitted to the Interventional Radiology Clinic of the University Hospital “Aretaieion” between April 2009 and May 2010 to undergo intra-arterial digital subtraction angiography. The study protocol was approved by the hospital's Ethics Commiteee (no. M-117 / 07-02-2008). The diagnosis of PVD was based on physical examination findings, clinical symptoms, measurement of the ankle- brachial index and duplex ultrasound scanning. Patients with recent cardiovascular or cerebrovascular event (myocardial infarction, cerebral infarction or hemorrhage), malignant disease, diabetes mellitus, renal failure or chronic inflammatory bowel disease (Crohn's disease or ulcerative colitis) were excluded from the study. Every other eligible patient was enrolled to the study. All study participants provided their written informed consent and they completed a questionnaire on demographic factors, eating habits and medical history. Height was measured by using a fixed stadiometer. Body weight was measured to the nearest hectogram. Fasting blood samples (20 ml venous blood) were collected prior to the angiographic examination. Fasting serum apolipoprotein (apoAI, apoAII, apoB, apoCII, apoCIII and apoE) concentrations were measured using Luminex xMAP immunoassay (Luminex Corp., Austin, TX, USA) 6-plex panel (Merch Millipore, MA, USA).

**Figure 1:**
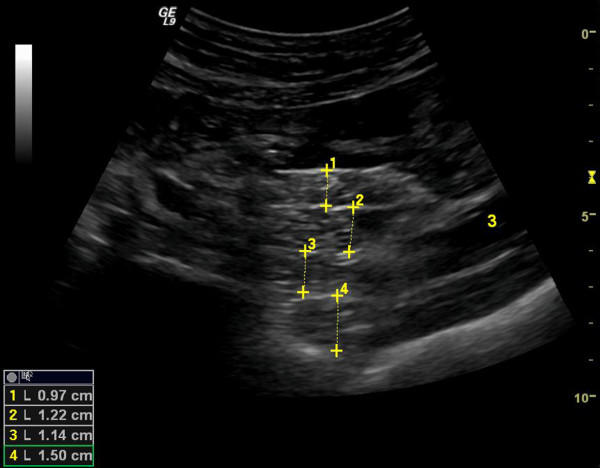
**Mesenteric fat thickness measurement/ detailed legend: mesenteric fat thickness estimated by ultrasonography. ** The mesenteric leaves are indicated by crosses.

On the same day, each participant was subjected to abdominal ultrasound scans by the same experienced radiologist, who was unaware of the subjects’ clinical and laboratory characteristics. The radiologist followed the methodology of Liu *et al.* for mesenteric fat estimation, using a 5–7 MHz linear-array transducer [13]. Briefly, the subjects were placed in a supine position and were asked to hold their breath during the measurements. The whole abdomen was scanned with emphasis in the periumbilical area, as more mesenteries are located in that area and can be easily identified. The mesenteric leaves appear as elongated structures with highly reflective peritoneal surfaces (Figure 1). Not all the mesenteries are visualized clearly during the examination, since some can be obscured by bowel gas. Usually, 6–10 measurements were performed during each ultrasound examination and the mean value of the three thickest mesenteric leaves was used for the analysis. A typical ultrasound examination would require 10–15 minutes by an experienced to the specific technique radiologist.

### Limitations of the study

The present study identified statistically significant correlations between MFT and serum apolipoproteins in PVD patients. However, future comparative studies using large numbers of patients and healthy subjects are necessary, in order to examine differences between subgroups and to derive useful further information. The use of fasting blood samples may have obscured a possible relationship of MFT and VLDL components. By performing single operator evaluations we avoided inter- but not intra-operator variability.

## Competing interests

Authors report no competing interests.

## Authors contributions

A.P. contributed to study design, data interpretation and drafted the manuscript, V.S. contributed to study design, performed ultrasonographic measurements and contributed to data interpretation, I.S.V: contributed to study design, performed the data analysis and drafted the manuscript, I.T. contributed to data analysis and interpretation, L.M.K contributed to data analysis and drafted the manuscript, P.K. contributed to data recording and analysis, I.D. contributed to data analysis and recording, I.I. contributed to data acquisition, I.K. contributed to data acquisition, D.G. contributed to data acquisition, C.K. contributed to study design and manuscript revision, D.N.P contributed to study design, data analysis, acquisition of data and revised the manuscript, A.H. contributed to study design, data analysis and manuscript revision. All authors read and approved the final manuscript.

## References

[B1] Deitel M The international obesity task force and "globesity" Obes Surg 2002 12 613 614 10.1381/096089202321019558 12448379

[B2] Kelly T Yang W Chen CS Reynolds K He J Global burden of obesity in 2005 and projections to 2030 Int J Obes (Lond) 2008 32 1431 1437 10.1038/ijo.2008.102 18607383

[B3] Must A Spadano J Coakley EH Field AE Colditz G Dietz WH The disease burden associated with overweight and obesity JAMA 1999 282 1523 1529 10.1001/jama.282.16.1523 10546691

[B4] Ohman MK Wright AP Wickenheiser KJ Luo W Eitzman DT Visceral adipose tissue and atherosclerosis Curr Vasc Pharmacol 2009 7 169 179 10.2174/157016109787455680 19356000

[B5] Vlachos IS Hatziioannou A Perelas A Perrea DN Sonographic assessment of regional adiposity AJR Am J Roentgenol 2007 189 1545 1553 10.2214/AJR.07.2366 18029899

[B6] Davidson MH Apolipoprotein measurements: is more widespread use clinically indicated? Clin Cardiol 2009 32 482 486 10.1002/clc.20559 19743499 PMC6653425

[B7] Scanu AM Edelstein C HDL: bridging past and present with a look at the future FASEB J 2008 22 4044 4054 10.1096/fj.08-117150 18716026 PMC2614615

[B8] Tian L Xu Y Fu M Jia L Yang Y Influence of apolipoproteinCII concentrations on HDL subclass distribution J Atheroscler Thromb 2009 16 611 620 10.5551/jat.1156 19729871

[B9] Kawakami A Yoshida M Apolipoprotein CIII links dyslipidemia with atherosclerosis J Atheroscler Thromb 2009 16 6 11 10.5551/jat.E607 19262004

[B10] Mahley RW Ji ZS Remnant lipoprotein metabolism: key pathways involving cell-surface heparan sulfate proteoglycans and apolipoprotein E J Lipid Res 1999 40 1 16 9869645

[B11] Perrini S Leonardini A Laviola L Giorgino F Biological specificity of visceral adipose tissue and therapeutic intervention Arch Physiol Biochem 2008 114 277 286 10.1080/13813450802334752 18946788

[B12] Ouchi N Ohashi K Shibata R Murohara T Adipocytokines and obesity-linked disorders Nagoya J Med Sci 2012 74 19 30 22515108 PMC4831247

[B13] Liu KH Chan YL Chan WB Chan JC Chu CW Mesenteric fat thickness is an independent determinant of metabolic syndrome and identifies subjects with increased carotid intima-media thickness Diabetes Care 2006 29 379 384 10.2337/diacare.29.02.06.dc05-1578 16443891

[B14] Bazzocchi A Filonzi G Ponti F Sassi C Salizzoni E Battista G Canini R Accuracy, reproducibility and repeatability of ultrasonography in the assessment of abdominal adiposity Acad Radiol 2011 18 1133 1143 10.1016/j.acra.2011.04.014 21724427

[B15] Liu KH Chan YL Chan JC Chan WB Association of carotid intima-media thickness with mesenteric, preperitoneal and subcutaneous fat thickness Atherosclerosis 2005 179 299 304 10.1016/j.atherosclerosis.2004.10.038 15777545

[B16] Liu KH Chan YL Chan JC Chan WB Kong WL Mesenteric fat thickness as an independent determinant of fatty liver Int J Obes (Lond) 2006 30 787 793 10.1038/sj.ijo.0803201 16418763

[B17] Liu KH Chan YL Chan WB Kong WL Kong MO Chan JC Sonographic measurement of mesenteric fat thickness is a good correlate with cardiovascular risk factors: comparison with subcutaneous and preperitoneal fat thickness, magnetic resonance imaging and anthropometric indexes Int J Obes Relat Metab Disord 2003 27 1267 1273 10.1038/sj.ijo.0802398 14513076

[B18] Derchi LE Solbiati L Rizzatto G De Pra L Normal anatomy and pathologic changes of the small bowel mesentery: US appearance Radiology 1987 164 649 652 3303119 10.1148/radiology.164.3.3303119

[B19] Ma RC Liu KH Lam PM Cheung LP Tam WH Ko GT Chan MH Ho CS Lam CW Chu WC Sonographic measurement of mesenteric fat predicts presence of fatty liver among subjects with polycystic ovary syndrome J Clin Endocrinol Metab 2011 96 799 807 10.1210/jc.2010-1608 21190980

[B20] Donnelly KL Smith CI Schwarzenberg SJ Jessurun J Boldt MD Parks EJ Sources of fatty acids stored in liver and secreted via lipoproteins in patients with nonalcoholic fatty liver disease J Clin Invest 2005 115 1343 1351 15864352 10.1172/JCI23621 PMC1087172

[B21] Pelletier-Beaumont E Arsenault BJ Almeras N Bergeron J Tremblay A Poirier P Despres JP Normalization of visceral adiposity is required to normalize plasma apolipoprotein B levels in response to a healthy eating/physical activity lifestyle modification program in viscerally obese men Atherosclerosis 2012 221 577 582 10.1016/j.atherosclerosis.2012.01.023 22321874

[B22] Despres JP Moorjani S Lupien PJ Tremblay A Nadeau A Bouchard C Regional distribution of body fat, plasma lipoproteins, and cardiovascular disease Arteriosclerosis 1990 10 497 511 10.1161/01.ATV.10.4.497 2196040

[B23] Ng TW Chan DC Barrett PH Watts GF Effect of weight loss on HDL-apoA-II kinetics in the metabolic syndrome Clin Sci (Lond) 2010 118 79 85 19456294 10.1042/CS20090110 PMC2782318

[B24] Krauss RM Siri PW Metabolic abnormalities: triglyceride and low-density lipoprotein Endocrinol Metab Clin North Am 2004 33 405 415 10.1016/j.ecl.2004.03.016 15158526

[B25] Meshkani R Adeli K Hepatic insulin resistance, metabolic syndrome and cardiovascular disease Clin Biochem 2009 42 1331 1346 10.1016/j.clinbiochem.2009.05.018 19501581

[B26] Yu YH Ginsberg HN Adipocyte signaling and lipid homeostasis: sequelae of insulin-resistant adipose tissue Circ Res 2005 96 1042 1052 10.1161/01.RES.0000165803.47776.38 15920027

[B27] Karagiannides I Pothoulakis C Neuropeptides, mesenteric fat, and intestinal inflammation Ann N Y Acad Sci 2008 1144 127 135 10.1196/annals.1418.009 19076372 PMC4404029

[B28] Bartolome N Rodriguez L Martinez MJ Ochoa B Chico Y Upregulation of apolipoprotein B secretion, but not lipid, by tumor necrosis factor-alpha in rat hepatocyte cultures in the absence of extracellular fatty acids Ann N Y Acad Sci 2007 1096 55 69 10.1196/annals.1397.070 17405916

[B29] Ng TW Watts GF Farvid MS Chan DC Barrett PH Adipocytokines and VLDL metabolism: independent regulatory effects of adiponectin, insulin resistance, and fat compartments on VLDL apolipoprotein B-100 kinetics? Diabetes 2005 54 795 802 10.2337/diabetes.54.3.795 15734858

[B30] Pittas AG Joseph NA Greenberg AS Adipocytokines and insulin resistance J Clin Endocrinol Metab 2004 89 447 452 10.1210/jc.2003-031005 14764746

[B31] Qin B Anderson RA Adeli K Tumor necrosis factor-alpha directly stimulates the overproduction of hepatic apolipoprotein B100-containing VLDL via impairment of hepatic insulin signaling Am J Physiol Gastrointest Liver Physiol 2008 294 G1120 G1129 10.1152/ajpgi.00407.2007 18372392

[B32] Arai T Yamashita S Hirano K Sakai N Kotani K Fujioka S Nozaki S Keno Y Yamane M Shinohara E Increased plasma cholesteryl ester transfer protein in obese subjects. A possible mechanism for the reduction of serum HDL cholesterol levels in obesity Arterioscler Thromb 1994 14 1129 1136 10.1161/01.ATV.14.7.1129 8018669

[B33] Kobayashi H Nakamura T Miyaoka K Nishida M Funahashi T Yamashita S Matsuzawa Y Visceral fat accumulation contributes to insulin resistance, small-sized low-density lipoprotein, and progression of coronary artery disease in middle-aged non-obese Japanese men Jpn Circ J 2001 65 193 199 10.1253/jcj.65.193 11266194

[B34] Panagiotakos DB Pitsavos C Yannakoulia M Chrysohoou C Stefanadis C The implication of obesity and central fat on markers of chronic inflammation: the ATTICA study Atherosclerosis 2005 183 308 315 10.1016/j.atherosclerosis.2005.03.010 16285994

